# Quality and Safety in Healthcare for Medical Students: Challenges and the Road Ahead

**DOI:** 10.3390/healthcare8040540

**Published:** 2020-12-04

**Authors:** Luz Berenice López-Hernández, Benjamín Gómez Díaz, Edgar Oswaldo Zamora González, Karen Itzel Montes-Hernández, Stephanie Simone Tlali Díaz, Christian Gabriel Toledo-Lozano, Lilia Patricia Bustamante-Montes, Norma Alejandra Vázquez Cárdenas

**Affiliations:** 1 Departamento de Calidad y Seguridad en la Atención Médica, Ciclo de vida, Universidad Autónoma de Guadalajara, Jalisco 45134, Mexico; kimontes@edu.uag.mx (K.I.M.-H.); stephanies.tlali@edu.uag.mx (S.S.T.D.); patricia.bustamante@edu.uag.mx (L.P.B.-M.); alejandra.vazquez@edu.uag.mx (N.A.V.C.); 2 Instituto Nacional de Rehabilitación, Ciudad de México 14389, Mexico; bngomez@inr.gob.mx; 3 Centro Universitario del Norte (CUNORTE), Universidad de Guadalajara, Guadalajara 46200, Mexico; edgar.zamora8148@academicos.udg.mx; 4 Facultad de Medicina, Universidad Nacional Autónoma de México, Ciudad de México 04510, Mexico; Christiantoledo@comunidad.unam.mx

**Keywords:** adverse event, COVID-19, safety and quality, medical students

## Abstract

**Background**: The development of skills, behaviors and attitudes regarding patient safety is of utmost importance for promoting safety culture for the next generation of health professionals. This study describes our experience of implementing a course on patient safety and quality improvement for fourth year medical students in Mexico during the COVID-19 outbreak. The course comprised essential knowledge based on the patient safety curriculum provided by the WHO. We also explored perceptions and attitudes of students regarding patient safety. **Methods**: Fourth year medical students completed a questionnaire regarding knowledge, skills, and attitudes on patient safety and quality improvement in medical care. The questionnaire was voluntarily answered online prior to and after the course. **Results**: In total, 213 students completed the questionnaires. Most students were able to understand medical error, recognize failure and the nature of causation, perform root-cause analysis, and appreciate the role of patient safety interventions. Conversely, a disapproving perspective prevailed among students concerning the preventability of medical errors, utility of reporting systems, just culture and infrastructure (*p* < 0.05). **Conclusion**: We found students had a positive perspective concerning learning quality in healthcare and patient safety during our course; nevertheless, their perception of the usefulness of reporting systems to prevent future adverse events and prevent medical errors is uncomplimentary. Medical education should promote error reporting and just culture to change the current perception of medical students.

## 1. Introduction

The current challenges of many healthcare institutions include rising healthcare costs, poor patient outcomes, shortage of health professionals, and the lack of continuity and integrated efforts for consolidating resilient health systems. The ability to retain quality healthcare despite such challenges is of utmost importance. The lack of understanding regarding team members’ roles, and miscommunication during the patient care process not only affect the quality of patient care but can also lead to serious adverse events [1]. Therefore, the way in which medical students understand their role in patient safety and healthcare quality may represent the beginning of a contributive effort to compete against current and future challenges of health systems.

**Figure 1:**
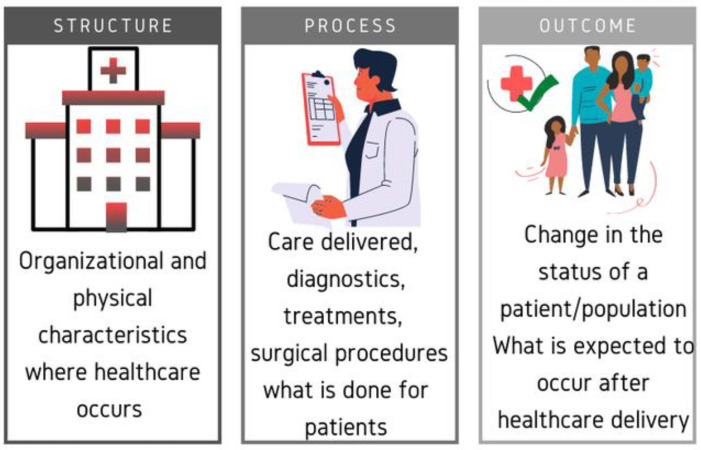
Donabedian healthcare quality model.

Based on Donabedian’s healthcare quality model, advances in the structure of care (equipment, instruments, supplies, standards, regulations, and procedures) should lead to improvements in clinical processes (counseling, medication, interaction with patients, families, and communities), which should in turn improve the outcome (certifications, recoveries, and patient satisfaction) (Figure 1) [2]. Consequently, reduction in unnecessary risks and preventable harm to a patient is expected during healthcare delivery. To achieve that, involvement of students in the prevention of adverse events and healthcare improvement should be promoted during medical education [3]. The development of skills, behaviors and attitudes regarding patient protection is of utmost importance for promoting safety culture for the next generation of health professionals [4,5,6]. Hence, with the aim of improving medical education, in 2010 the WHO established a patient safety curriculum (PSC) for medical schools [7]. Nevertheless, it remains challenging to establish how the subject should be taught and how the preparedness of medical students is conceived to improve patient safety in different settings [4,8,9,10,11]. In Mexico, about 8% of hospitalized patients experience adverse events, 2% of such events result in the patient’s death. Approximately 62% of the adverse events were preventable [12]. Herein, we describe our experience with the implementation of a course on quality and safety in healthcare (QSH) for medical students. The objective of implementing this course was to help undergraduates to develop skills and acquire basic expertise that guarantees professional performance in their medical practice regarding QSH. The course comprised essential knowledge for students before they began undergraduate internal training in hospitals (UIT). Due to the scarcity of information regarding perceptions and attitudes of undergraduate medical students on QSH in Mexico and with the aim of improving our recently implemented course, we focused our attention on fourth year students at the same university. The questionnaire was answered before the course and after finishing it, during the quarantine derived from the COVID-19 outbreak in Mexico.

## 2. Materials and Methods

### 2.1. Course Content

Students spend 13 h per week for two weeks, distributed in sessions of three hours per day, one lecture session (duration 1 h) and a two-hour session, on problem-based learning (PBL), brainstorm and team-building activities. We adapted teaching materials to the eleven topics suggested by the WHO: (1) what is patient safety? (2) What is human factors engineering, and why is it important to patient safety? (3) Understanding systems and the impact of complexity on patient care, (4) being an effective team player, (5) understanding and learning from errors, (6) understanding and managing clinical risk, (7) introduction to quality improvement methods, (8) engaging with patients and caregivers, (9) minimizing infection through improved infection control, (10) patient safety and invasive procedures and (11) improving medication safety [7]. In addition to the WHO topics, this course included information regarding national and international accreditation/certification requirements for hospitals and the national agreement called “Essential Actions for Patient Safety”, which is mandatory for all members of the National Health System, as it was declared by the Consejo de Salubridad General (Mexican health authority with normative, advisory, and executive functions). A syllabus including course policies, rules, and regulations, required texts, and a schedule of assignments for this course was generated for the first time including 10% daily assessments, 20% final exam, 10% teamwork and 60% corresponded to problem-based learning (PBL) exercises.

### 2.2. Participants and Procedures

Participants were students from Universidad Autónoma de Guadalajara, which is a private university with presence in two states of Mexico (Tabasco/Southeast and Guadalajara/Northwest). Nonprobability sampling was used to include participants.

Fourth year medical students completed a newly implemented course on QSH, the course was focused on providing basic knowledge and preparation for the activities that students usually perform during their internship, which begin a few months after finishing the course. To explore the perceptions and attitudes regarding QSH, all the students taking the course were invited to respond to a questionnaire prior to and after the course. The questionnaire was voluntarily answered online by registering e-mails and no other identification data. Only duplicated answers to the questionnaire were excluded from the study.

### 2.3. Ethical Approval

This study was registered and approved by our institutional research coordination. Participation was voluntary and the answers from individuals were anonymized for research purposes. The number assigned to this project by local committees was 20-1307-04 (reference).

### 2.4. Instruments 

#### 2.4.1. To Evaluate Perceptions and Attitudes of Students

**Figure 2:**
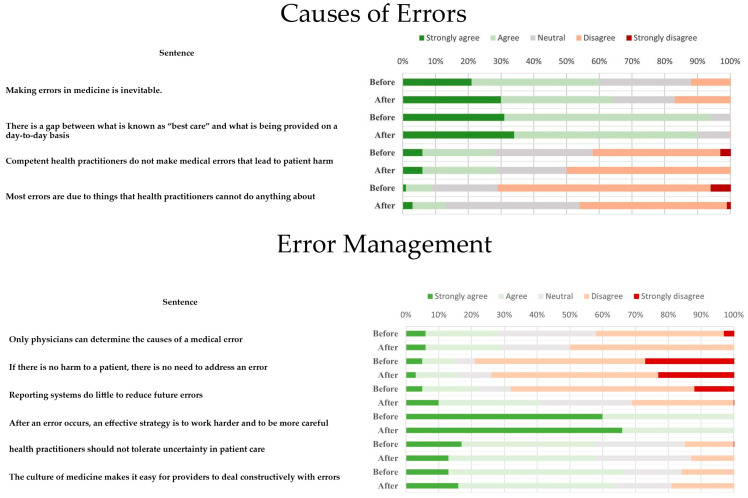
Pre-test and post-test answers.

A five-point Likert scale (5 = strongly disagree, 4 = disagree, 3 = neutral, 2 = agree, 1 = strongly agree) was used to measure the perceptions and attitudes of the students. The questionnaire was adapted from the studies of Madigosky and Nabilou et al. [8,9] (Figure 2). 

#### 2.4.2. To Evaluate Students’ Knowledge Concerning the Course and to Get Insights into the Regional Environment Perception of Students

Daily assessments of five questions were used to evaluate students’ progress about concepts such as safety, quality, and error. In addition, problem-based learning (PBL) was applied as a strategy to promote students’ skills to analyze “real world” situations. We selected cases from public information available through local media. At the end of the course, to explore students’ skills we asked them to analyze cases focusing on the current pandemic as their clinical scenario. We asked them to answer five specific questions after the course (with no curricular value) (Table 1).

### 2.5. Data Analysis

The non-parametric paired samples Wilcoxon test was used to compare paired data derived from pre- and post-questionnaires. A chi-square analysis test was performed for gender to explore significant differences of categorical variables. SPSS statistical package was used for data processing.

## 3. Results

During the course, we reviewed essential concepts covered by topics suggested by the WHO, but we also discussed how preventing avoidable adverse events in a regional context should be pursued. We additionally explored perceptions and attitudes of students regarding patient safety. In total, 213 out of 241 students (88% response rate) completed the questionnaires, 131 (61.5%) were female and 82 (38.5%) were male. To get insights into changes derived from our course, 107 out of 213 completed the pre-test and post-test questionnaire (50.2% response rate). In this study, patient safety was considered an important topic among students (12.2% strongly agree, 87.8% agree), they were also willing to receive more information on patient safety (58% strongly agree/agree, 25% neutral, 17% disagree/strongly disagree) and students felt skilled enough to analyze contributing factors when an adverse event occurs through cause-root analysis (56% strongly agree/agree, 27% neutral, and 17% disagree/strongly disagree). Responses to teaching items regarding patient safety disclosed positive attitudes among students in half of the questions, most of them agreed that patient safety is an important topic, they felt capable of analyzing an adverse event to find the causes and contributing factors (root-cause analysis, Ishikawa diagram), they felt willing to help a peer when an error occurs and agreed to receive more information on patient safety issues. Nevertheless, less students agreed to answers such as “Physicians should routinely spend part of their professional time working to improve patient care”, “Physicians should routinely report medical errors, learning how to improve patient safety is an appropriate use of time in medical school and disclosing an error to a faculty member”; this was not different between groups (Table 2). Regarding the topic “Causes of Errors”, the sentence “*Making errors in medicine is inevitable*” showed a difference between male and female groups (*p* = 0.04). In general, an adverse perspective of the occurrence of medical errors was prevalent (Table 3. This perspective did not change after the completion of the course, as no difference was revealed by the Wilcoxon test (Figure 2). Interestingly, the pre-test and post-test answers of two questions: “*Reporting systems do little to reduce future errors*” and “*Most errors are due to things that health practitioners cannot do anything about*” showed significant change towards a negative perspective of students (*p* < 0.05). Concerning questions focused on regional problems of Mexico on patient safety and quality in healthcare, most students perceived structure as the worst element of quality in Mexico, according to Donabedian’s model (Figure 1). A great proportion of student’s answers correctly classified an infection-related sentinel event and mentioned the quality dimension proposed by the IOM (Institute of Medicine, USA) applicable to such case.

## 4. Discussion

Currently, the COVID-19 outbreak threatens health systems around the world as medical doctors at the front line of the pandemic suffer infection risk, pressure, and work overload, which not only impacts on one’s well-being but also patient safety and functioning of the healthcare system. Indeed, the current pandemic has upended medical education around the world, and some countries have mobilized students in medical, nursing, and other health education programs nearing the end of their studies to provide support for patients and frontline physicians in tasks that do not require exposure to SARS-CoV-2 [13,14]; therefore, the role of medical students in preventing patient harm and specially ameliorating the burden of the current pandemic is crucial for health systems [15]. Strengthening skills and knowledge regarding quality and safety in healthcare for students implies the understanding that applicable learning in healthcare occurs in settings with complex interactions such as technology, social media influence, policies, procedures, resources, and the disease process itself, which in some cases result in adverse events. Therefore, medical students at the end of their preparation must be able to act optimally in high-stress situations, in which decision-making is of ultimate importance to cope with the healthcare needs. In developing countries such as Mexico, medical students are witnesses and are even directly involved in unsafe situations and deficient and inconsistent care, in part because of changes in the National Health System [16] and new factors add more complexity to the current situation. Unlike other countries, a wave of violence against healthcare workers has been experienced in Mexico, as healthcare professionals have been wrongly accused of spreading the disease. It has been reported that people have purposely cough or spit on healthcare workers [17]. In addition, the healthcare workers’ risk of dying in Mexico is four times higher than in the United States, and eight times higher than in Brazil [18]. This scenario is what our students will face in a few months.

UIT students, apprentices in social service and medical residents represent an essential part of the healthcare personnel, making up approximately 58,972 medical doctors in the clinical phase of the medical education system [14]. They carry out learning-related tasks and might be exposed to patients with COVID-19. Therefore, as of 24 March 2020, it was decided that all undergraduate medical students in Mexico should not be present in COVID-19 risk areas, except for medical interns in social services and graduated medical residents, because they have the dual status of student/worker-employee, and then they must receive the required equipment for protection when treating suspected and confirmed COVID-19 patients [14]. It has been recently described that major contributors to unsafe patient care in low-to middle income countries are inadequate facilities, shortage of basic equipment, insufficient healthcare supplies, poor hygiene/sanitation, overcrowding and understaffing [19], which agree with the perceptions of students reported herein (Table 1).

Our findings suggest that medical education should promote error reporting and just culture to change the current perception of medical students, since a negative perspective in this regard was detected (Supplementary Materials). Interestingly, this is consistent with other studies in Mexico, in which both medical residents and interns consider that errors are used against their academic records (30% and 50%, respectively) [20]. Students’ perception of the occurrence of errors and the thought that nothing can be done to avoid them may be related to the perception that Mexico has inferior infrastructure and there is little that healthcare professionals can do about it [21,22]. This is also concordant with the fact that less students agreed with spending time on patient safety issues, because it is perceived by the participants of this study that the main problems of healthcare in Mexico are derived from the infrastructure crisis, comprising inadequate facilities and insufficient healthcare supplies, that risks both the safety of patients and students independently of the knowledge, performance, will and attitudes of medical trainees/personnel. Furthermore, awareness could also be focused on how to promote non-punitive culture when an error occurs, especially in trainees. It was observed in the cases reviewed for PBL that the informative style, the redaction, and the images presented in the news correspond to a punitive culture rather than an informative and truthful exercise of freedom of expression. Therefore, the road ahead to pursue the next generation of optimally educated healthcare professionals may require a set of efforts by medical schools, students, and the government, but also the community and the forms of communication that allow us to interact as a society to face the challenge of improving healthcare.

### 4.1. Strengths and Limitations

A strength of this study is the acceptable sample of respondents (88% general, 50.2% for pre-test and post-test questionnaire). In addition, our results underline the perceptions of the students regarding patient safety issues just before they enter hospitals for intensive training and after reviewing cases and having received the course. A limitation was that respondents were not totally anonymous when responding questioners, since the questionnaire was online and e-mails were registered when participating, and this may affect students’ answers. Another disadvantage was that not all the students answered pre- and post-questionnaires. Despite these limitations, we believe that our study adds to the current knowledge regarding the perception of students on quality and safety in healthcare issues in México.

### 4.2. Suggestions for Further Research

To begin with, this study identified areas for development to enhance patient safety. Strengthening of safety culture ought to be a priority in our program to change the perception and attitudes of students. Main issues to improve on among students are communication openness and non-punitive response to error and the role of reporting systems in preventing future errors. We recommend further research that includes more universities to extend our perspective of preparedness of medical students regarding safety and quality in healthcare.

## 5. Conclusions

We found evidence of willingness and knowledge of students concerning learning quality in healthcare and patient safety during our course; nevertheless, their perception of the usefulness of reporting systems to prevent future adverse events and prevent medical errors is uncomplimentary. Students have a disapproving perspective of the current situation of Mexico’s health infrastructure.

## Figures and Tables

**Table 1:** Regional focused questions regarding safety and quality in healthcare.

Question	Most Plausible Answer (Percentage)	Other Answers (Percentage)
According to the dimensions of the quality of the IOM, which would be awarded the first place in importance at this moment (pandemic) to avoid the collapse of the health system?	Efficiency (39.1%)	Safety (0.45%), effectiveness (32.2), timeliness, (6.1%), patient-centered (21.7%), and equitable (0.45%).
According to the Avedis Donabedian model of quality, the lack of mechanical ventilators to fulfill the needs of the current pandemic corresponds to…	Infrastructure-equipment (95.5%)	Process-environment (2.6%) Process-management (0.9%)
In which of the elements of the Avedis Donabedian quality model do you think Mexico is worst?	Structure (53.9%)	Process 27.8% Results 18.3%
In which of the elements of the Avedis Donabedian quality model do you think Mexico is best?	Process (47%)	Structure (26%) Results (27%)
If a patient enters the emergency room for acute abdominal pain and derived from the current health contingency, acquires COVID-19 infection in the hospital and dies of respiratory complications, what type of event would be?	Sentinel (72.2%)	Adverse event (25.2%) Near miss (2.6%)

**Table 2:** Students’ responses to teaching items of patient safety education and skills.

Teaching Items of Patient Safety Education and Skills							
Sentence	Students	Strongly Agree	Agree	Neutral	Disagree	Strongly Disagree	*p*-Value
Physicians should routinely spend part of their professional time working to improve patient care	Female	29	1	3	40	0	0.84
	Male	19	0	2	20	0	
Patient safety’ is an important topic	Female	8	65	0	0	0	0.38
	Male	6	35	0	0	0	
Physicians should routinely report medical errors	Female	31	1	2	39	0	0.11
	Male	14	2	1	24	0	
Learning how to improve patient safety is an appropriate use of time in medical school	Female	32	3	3	34	1	0.60
	Male	25	1	3	12	0	
Would like to receive further teaching on patient safety	Female	34	32	3	3	1	0.22
	Male	12	25	3	1	0	
Supporting and advising a peer who must decide how to respond to an error	Female	37	6	20	9	1	0.45
	Male	22	1	9	9	0	
Analyzing a case to find the cause of an error	Female	37	6	20	10	0	0.46
	Male	20	1	11	9	0	
Disclosing an error to a faculty member	Female	36	1	3	33	0	0.84
	Male	23	0	2	16	0	

**Table 3:** Overview of students’ perceptions regarding causes of error and management.

**Causes of Errors**							
**Sentence**	**Group**	**Strongly Agree**	**Agree**	**Neutral**	**Disagree**	**Strongly Disagree**	***p*-Value**
Making errors in medicine is inevitable.	Female	31	35	26	34	5	0.04
	Male	28	24	9	13	8	
There is a gap between what is known as “best care” and what is being provided on a day-to-day basis	Female	37	65	12	16	1	0.49
	Male	26	40	9	5	2	
Competent health practitioners do not make medical errors that lead to patient harm	Female	6	22	26	68	9	0.43
	Male	6	14	15	36	11	
Most errors are due to things that health practitioners cannot do anything about	Female	1	7	30	73	20	0.37
	Male	2	8	23	37	12	
**Error Management**							
**Sentence**	**Students**	**Strongly Agree**	**Agree**	**Neutral**	**Disagree**	**Strongly Disagree**	***p*-Value**
Only physicians can determine the causes of a medical error	Female	9	23	28	57	14	0.57
	Male	8	18	13	31	12	
If there is no harm to a patient, there is no need to address an error	Female	2	13	14	61	41	0.72
	Male	2	7	5	44	24	
Reporting systems do little to reduce future errors	Female	12	24	28	51	16	0.10
	Male	2	22	12	31	15	
After an error occurs, an effective strategy is to work harder and to be more careful	Female	80	46	3	2	0	0.22
	Male	45	30	1	5	1	
health practitioners should not tolerate uncertainty in patient care	Female	9	51	30	35	6	0.21
	Male	12	25	14	25	6	
The culture of medicine makes it easy for providers to deal constructively with errors	Female	22	67	19	19	4	0.51
	Male	20	34	10	15	3	

## Supplementary Materials

The following are available online at https://www.mdpi.com/2227-9032/8/4/540/s1. Supplementary 1, Original dataset and supplementary video abstract.
